# The Association between Mental Wellbeing, Levels of Harmful Drinking, and Drinking Motivations: A Cross-Sectional Study of the UK Adult Population

**DOI:** 10.3390/ijerph15071333

**Published:** 2018-06-25

**Authors:** Anita Appleton, Rosie James, John Larsen

**Affiliations:** Research and Impact Team, Drinkaware, London EC2M 5QQ, UK; rjames@drinkaware.co.uk

**Keywords:** alcohol consumption, drinking motivations, mental health, well-being, United Kingdom

## Abstract

Mental well-being and excessive alcohol consumption each represent a significant public health concern, and evidence suggests an association between them. Furthermore, drinking motivations associated with harmful drinking have been studied, but not systematically in the UK population. A representative sample of 6174 UK adults aged 18–75 were surveyed online. Low risk drinkers were found to have higher mental well-being than hazardous, harmful, and, probable, dependence drinkers. Using a hierarchical multiple regression analysis, it was found that just over 5% of the variance in well-being scores was accounted for by the level of harmful drinking and drinking motivation; the most significant contribution was drinking to cope. Among people drinking to cope, those drinking in more harmful ways were statistically significantly more likely to have low well-being compared to less harmful drinkers. In the UK adult population there is a clear association between poor mental well-being and harmful drinking. Furthermore, coping was a significant motivation to drink for many with low mental well-being. While mental well-being was found to be directly linked with levels of harmful drinking, the motivation for drinking was a stronger predictor of mental well-being.

## 1. Introduction

With alcohol being a leading cause of death worldwide it is a considerable public health concern [1], and there is clear evidence of a link between alcohol misuse and mental health and well-being within a variety of different populations [2,3,4,5,6]. There is also a comorbidity between harmful drinking and diagnosed mental illnesses, such as depression [3] and anxiety [7].

Hazardous alcohol intake has been found to be associated with low mental well-being [2,8,9,10,11]. The term “mental well-being” denotes positive mental health and emotional well-being [12]. It refers to the mental state of an individual, how they are feeling, and their ability to cope with day-to-day stressors; importantly, it does not depend on the presence or absence of a diagnosed condition [12]. Specifically, in this paper, we adopt a definition of mental well-being focusing partly on self-realization and defining it in terms of the degree to which a person is fully functioning, and partly regarding pleasure attainment (or happiness) and pain avoidance. These are also known, respectively, as *eudaimonic* and *hedonic* aspects of mental well-being [13,14]. Mental well-being is increasingly recognized, along with other non-clinical terms, such as “happiness” [15], as being significant in the consideration of a population’s health and well-being [16], and it is of particular interest to behaviour change efforts aiming to improve lifestyles in a population [17].

Internationally, a body of research is emerging on mental well-being and alcohol consumption. One study from New Zealand found that there was a negative correlation between aggregate population eudaimonic well-being and alcohol consumption, and this correlation was strengthened as drinking became more problematic [11]. The researchers concluded that low risk drinking affected aggregate happiness positively, whereas high risk drinking affected aggregate happiness negatively [11]. A Finnish study found that binge-drinking and hazardous drinking was associated with poorer eudaimonic and hedonic well-being. Light to moderate drinking was found to have no relation to poor mental well-being; however, this provided a threshold, after which the amount drunk was strongly associated with poor mental well-being [10].

A study in England looking at the relationship between alcohol and well-being found that abstainers and heavy drinkers were more likely to experience low well-being [6]. The study found that consuming moderate levels of alcohol (≤32 grams of alcohol/day/men or ≤24 grams of alcohol/day/women) was linked to higher mental well-being when compared to people who drank more or abstainers [2]. Furthermore, another study in England demonstrated a bidirectional causal relationship between levels of alcohol consumption and mental well-being [3]. In a study looking at adults in North West England, it was found that higher risk drinkers (more than 400 grams of alcohol per week for males and more than 280 for females) displayed lower mental well-being than all other categories of drinkers. The highest mental well-being score was among low risk drinkers [4].

It has been suggested that the link between high levels of alcohol consumption and the mental health conditions of anxiety and depression can, at least in part, be explained by “self-medication” [18], whereby people are seeking to reduce their symptoms by drinking, although this leads to a negative spiral of increasingly worse mental health symptoms and higher levels of drinking [19,20]. While there has been some research to investigate how the association between anxiety and excessive drinking is influenced by drinking motivations [21], this has been restricted to younger people, often college students. This research suggests that particular issues related to these younger drinkers should be considered. For example, a study of 13–16 year olds emphasized the importance of considering social motives as predicting excessive drinking [22], whereas a study among college students suggested that those who drink to cope with social anxiety are more likely to drink excessively and that interventions could be developed to help build coping skills in this population group [23]. 

Furthermore, some studies on the association between alcohol consumption and mental well-being have identified an “abstainer effect”, where abstainers were found to be more likely to experience lower well-being and higher levels of distress than low risk or moderate drinkers [24]. The abstainer effect linked to mental health has been attributed partly to the “sick-quitter hypothesis”, which, in the context of alcohol consumption, suggests that adverse outcomes relating to abstainers can, in part, be explained by the proportion among these who were previously heavy drinkers [24,25]. In addition, the social relations hypothesis suggests that abstainers have a propensity toward poorer social relationships compared to moderate drinkers, thus, providing an explanation for their poorer mental health [25]. The abstainer effect has been reported within older age groups [24]; however, the literature is inconsistent, most likely due to the use of different measures for well-being [26].

These studies examining drinking motivations, mental health, and levels of alcohol consumption offer valuable insights to inform the development of targeted interventions and messages. However, to our knowledge, there is no published research examining motivations for drinking in a representative, adult UK drinking population, and linking this to aspects of mental well-being and levels of harmful drinking. This study aims to understand these interrelations, which may help inform efforts both to identify individuals who could be more effectively supported to reduce their drinking through higher mental well-being, and to engage individuals to take steps to reduce their alcohol consumption through the prospect of experiencing higher well-being.

## 2. Materials and Methods

Data was used from the 2017 Drinkaware Monitor survey, which investigated drinking behaviours, occasions and motivations, harmful drinking behaviours, and moderation techniques across the UK [27]. The survey was conducted on behalf of Drinkaware by YouGov, a market research and data analytics firm. The survey comprised a representative sample of 6174 UK adults aged 18 to 75 who were surveyed online in March and April 2017. The sample was adjusted by weighting to match UK population levels according to age, gender, region, and social grade; with quotas based on census information. YouGov approached participants who are members of their online panel via email using an initial generic email invitation inviting participants to take part in the survey. Detailed information on the study was contained on the introduction and consent page.

The following standardized tools included in the survey were used in the analysis for this study.

### 2.1. Alcohol Use Disorder Identification Test (AUDIT)

The AUDIT tool was developed by the World Health Organization [28], with the purpose of screening for an individual’s level of risk and/or harm in relation to their alcohol consumption patterns. It is a useful tool in identifying some specific consequences of harmful drinking and dependence on alcohol. The test contains 10 questions, each of which is scored between 0–4, where 0 indicates “never” or “no” and 4 represents an increased likelihood of risky drinking. Completion of the tool results in a summed score between 0 and 40. Scores for this tool have been grouped, as shown in Table 1. The AUDIT was used to determine participants’ harmful drinking category.

### 2.2. The Short Warwick-Edinburgh Mental Wellbeing Scale (SWEMWBS)

The Warwick-Edinburgh Mental Wellbeing Scale (WEMWBS), developed by Warwick and Edinburgh Universities, was developed as a way to assess mental well-being in the general population [29]. The WEMWBS uses a set of 14 positively worded statements about specific thoughts and feelings, with five response categories to determine how often an individual has experienced them. A shortened version of this tool, the SWEMWBS, uses seven of the WEMWBS questions [30]. The SWEMWBS was used in the survey to allow for analysis of any differences in response patterns according to mental well-being [31]. Each of the seven items is scored on a five point response scale, where 1 signifies “none of the time” and 5 signifies “all of the time”. This results in individual participants receiving an overall score between 7 and 35, with a lower score denoting a lower level of mental well-being. For the analysis in this report, the level of mental well-being has been assessed as either low, medium, or high (Table 2). Low scores were calculated as at least one standard deviation below the mean and high scores as at least one standard deviation above the mean [32].

### 2.3. The Drinking Motive Questionnaire: Revised Short Form (DMQ-R SF)

The Drinking Motive Questionnaire: Revised Short Form (DMQ-R SF) [21] consists of 12 motivations for drinking questions and provides participants with a five point response scale, where 1 indicates “almost never/never” and 5 indicates “almost always/always”. The questionnaire is used to determine the extent to which an individual drinks for conformity reasons (e.g., to be liked), coping reasons (e.g., to improve a bad mood), enhancement reasons (e.g., because they like the feeling), and social reasons (e.g., to improve parties and celebrations). If a participant has selected at least one of the subscale questions as a drinking motivation, we refer to them as drinking for that motivation. For example, coping drinkers refer to anyone who drinks for at least one of the reasons in the coping subscale questions.

### 2.5. Sociodemographic Information

Participants were asked to indicate their age, gender, education, and occupation. Age was calculated from the participant’s date of birth. Education level was calculated from a list where participants were asked to choose their highest completed education. Social grade was calculated based on the occupation (or pension situation) of the chief income earner in the household.

### 2.6. Hypotheses and Analysis Strategy

The data used in this study was analysed using IBM SPSS Statistics version 22.0 (IBM, Armonk, NY, USA) [33]. Statistical significance was set at the 0.05 level. 

The first hypothesis is that harmful drinking is significantly related to mental well-being. Relative percentages are reported for the categorical variables included within the analysis and are reported in the cross-tabulation (Table 3). The significance of the relationship between categories of harmful drinking (AUDIT) and well-being categories (SWEMWBS) was calculated using a two-proportion z-test (pooled for H0: p_1 = p_2) with a Bonferroni correction. 

To test the second hypothesis, that there is a relationship between well-being, drinking motivations, and harmful drinking in the UK population, a chi-squared statistical test was used to test the significance of the relationship between the categorical variables of the harmful drinking category and drinking motivation (Table 4). A hierarchical multiple regression analysis was run using the raw scores, rather than the categories, to control for demographic variables and test the amount of variation that each predictor (demographic data, AUDIT score, and drinking motivation) contributed to the overall SWEMWBS score (Table 5). Lastly, relative percentages are reported for the categorical variables of drinking motivation and well-being, split by AUDIT risk group, and are reported in the cross-tabulation (Table 6). A two-proportion z-test (pooled for H0: p_1 = p_2) was used to test the significance of the relationship between harmful drinking category and well-being category by each drinking motivation (Table 6). For Table 1, Table 2, Table 3 and Table 4 and Table 6, categories were used to discuss AUDIT scores within the allocated groupings and in Table 5 raw scores were used for the hierarchical multiple regression model. The categories are groupings of the raw scores and, therefore, should not be considered as separate variables.

## 3. Results

Harmful drinking is significantly related to low well-being. As can be seen by the frequencies cross tabulated in Table 3, there is a significant relationship between harmful drinking categories and well-being categories, X^2^ (8) = 65.203, *p* < 0.001, with a very small effect size (V = 0.073). 

Low risk drinkers are significantly more likely to have higher well-being (14.1%) than hazardous (9.4%) and harmful (7.3%) drinkers. Whilst 14.7% of low risk drinkers have low mental well-being, this rises to 27.3% among probable dependence drinkers. However, abstainers are less likely to have high well-being (11.7%) compared to low risk drinkers, yet they are more likely to have higher well-being than hazardous and above drinkers (Table 3).

There is a relationship between mental well-being, drinking motivations, and harmful drinking in the UK population. Motivations for drinking were grouped into four categories within the dataset: Coping reasons, conformity reasons, social reasons, and enhancement reasons. Findings show that the largest difference between mental well-being categories is between those who drink for coping reasons, followed by those who drink for conformity reasons. Almost three quarters (72.8%) of those with low mental well-being drank for coping reasons, compared to 35.1% of those with high mental well-being. Over half (53.1%) of those with low mental well-being drank for conformity reasons, compared to 33.2% of those with high mental well-being. 

Table 4 depicts the relationship between drinking motivation and mental well-being, and shows that as mental well-being increases, the proportion that drink to cope (X^2^ (2) = 222.352, *p* < 0.001, V = 0.203) or to conform (X^2^ (2) = 63.274, *p* < 0.001, V = 0.108) decreases. The relationship is not as strong, demonstrated by the small effect sizes, but it is also significant for those who drink for social (X^2^ (2) = 26.339, *p* < 0.001, V = 0.070) or enhancement (X^2^ (2) = 16.933, *p* < 0.001, V = 0.056) reasons.

To understand how much of the variance in the raw SWEMWBS score is accounted for by particular variables, a hierarchical multiple regression analysis was run (excluding cases pairwise). There were no violations of assumptions. In the first step of the hierarchical multiple regression, four control predictors were entered based on previous research demonstrating their relationship with well-being: gender, age, education level, and social grade [32,34,35]. This model was statistically significant (F (4, 5384) = 77.211, *p* < 0.001) and explained 5.4% of the variance in the well-being score. Age, education level, and social grade all made a significant unique contribution to the model, but gender did not (see Table 5). 

In the second step, the raw AUDIT score variable was entered and the total variance explained by the model as a whole was 6% (F (5, 5383) = 68.687, *p* < 0.001). The introduction of the AUDIT score explained an additional 0.6% of the variance in the well-being score, after controlling for demographic variables (R^2^ change = 0.006; F (1, 5383) = 32.767, *p* < 0.001). 

Drinking motivation was added in the third step and the total variance explained by the model as a whole was 10.5% (F (9, 5379) = 70.130, *p* < 0.001). Taking into consideration drinking motivations explained an additional 4.5% of the variance in the well-being score after controlling for demographic variables and the AUDIT score (R^2^ change = 0.45; F (4, 5379) = 67.681, *p* < 0.001).

In the final adjusted model, seven out of the nine predictor variables were statistically significant, with coping reasons for drinking recording a higher Beta value (β = 0.228, *p* < 0.001) than the other predictor variables. 

When looking at the proportion of people in each harmful drinking category and drinking for different reasons by mental well-being category (Table 6) we see that as harmful drinking increases, the proportion of those with lower well-being also increases; we can see this more granularly by looking at the drinking motivations in each harmful drinking category. A two-proportion z-test (pooled for H0: p_1 = p_2) with a Bonferroni correction showed the statistically significant differences between each harmful drinking category, for example, low risk and hazardous coping drinkers were less likely to have low well-being (18.8% and 19.9%, respectively), compared to harmful (24%) and probable dependence (28.7%) coping drinkers at the .05 significance level (Table 6).

These findings suggest that, while drinking to cope is a significant contributor to low mental well-being, this contribution is accentuated when divided by the harmful drinking category. Among those who drink to cope, more harmful drinkers are statistically significantly more likely to have low well-being compared to less harmful drinkers.

## 4. Discussion

This study has examined whether harmful drinking is significantly related to low mental well-being and investigated the relationship between harmful drinking, mental well-being, and drinking motivation within the UK population. The results are in line with the wider literature, finding low risk drinkers are significantly more likely to have higher well-being than those who drink at hazardous, harmful, and probable dependence levels. However, confirming findings from other research [4], although abstainers were found to be less likely to have high well-being compared to low risk drinkers, they were more likely to have higher well-being than hazardous, harmful, and probable dependence drinkers, supporting the theory of an “abstainer effect” [24].

Around one in seven (14.1%) low risk drinkers report high mental wellbeing in contrast to one in fourteen (7.3%) harmful drinkers and less than one in ten hazardous and probable dependence drinkers (9.4% and 9.3%). Similarly, low mental well-being is more prevalent the more harmful the drinking is, rising from 14.7% among low risk drinkers to more than one in four (27.3%) among probable dependence drinkers. These findings are supported by previous literature, which has found that binge drinking, hazardous drinking, and high-volume drinking are associated with poorer mental health [10]. 

Our examination of how mental wellbeing and drinking motivations are interlinked in the UK adult population showed that the largest difference between mental well-being categories was between those who drink to cope followed by those who drink to conform. Among people with low mental well-being, almost three in four (72.8%) drank to cope, compared to just over a third (35.1%) of those with high mental well-being. The finding that a large proportion of those with low mental well-being drink for coping reasons are in line with previous research examining the link between low mood and drinking to cope [23]. By examining well-being in relation to conformity motivations for drinking, it was found that over half (53.1%) of those with low mental well-being drank for conformity reasons, compared to a third (33.2%) of those with high mental well-being. It was a general finding that those with high mental well-being were, overall, less likely to drink for any reason, with close to one in four indicating not to be drinking for social or enhancement reasons (both at 23%), which are considered the “positive” reasons. In contrast, only between one in six and one and seven (15–18%) people with low or medium mental well-being drink for neither of these reasons. This finding could be understood, at least in part, as support for the “self-medication” theory stating that people may use drinking as a tool to either cope with difficult issues or enhance desired emotional states [18]. It suggests that higher levels of mental well-being are associated with a decreased use of drinking to self-medicate, and this difference is most pronounced in respect to people with low mental well-being who are more likely to use the self-medication strategy to manage negative situations or emotional states (coping and conformity reasons).

Uniquely, the study examined the extent to which a range of factors predicted mental well-being. It was found that, while the level of harmful drinking explained some of the variance (0.6%), the single biggest predictor of well-being was a person’s drinking motivation (4.5%). In addition, it was found that age, education level, and social grade combined explained just over 5% of the difference in mental well-being, whereas gender was found not to have any effect. This finding shows that, although mental well-being is directly linked with levels of harmful drinking, the motivation for drinking is a stronger predictor of mental well-being.

Possible implications of these findings are that understanding people’s motivations for drinking should be a key consideration in efforts not only for reducing harmful drinking, but also to improve their mental well-being. In particular, alcohol may be used as “self-medication” to cope with low mental well-being, and it should be considered whether alternative support and guidance could be provided to reduce the incidence of harmful drinking resulting from this.

A key strength of this study is the large sample size (over 6000) offering a representative sample of the UK population aged 18–75 and the use of standardized and validated tools to measure harmful drinking (AUDIT), mental well-being (SWEMWBS), and drinking motivations (DMQ-R SF). This has allowed a detailed investigation of the interrelation between these factors, adding to the literature in the field, and, potentially, informing future alcohol harm reduction interventions. 

Limitations of the study mainly relate to the study methodology, being an online panel survey relying on self-selection and self-completion. Although issues of representativeness have been adjusted for in the analysis through weighting the data according to age, gender, region, and social grade, it has been suggested that using alternative methodologies may more effectively engage hard-to-reach groups (e.g., people in transient accommodation) [36]. Although the privacy of the online methodology may support more honest self-reporting of drinking habits, the methodology is likely to lead to underreporting of what is perceived as socially undesirable behaviours. It has been highlighted in other research that considering “special event” drinking can offer a more accurate assessment [37]. With these limitations in mind it is fair to assume that harmful drinking is likely to be underreported in the present study.

## 5. Conclusions

To our knowledge, this is the first time a study has looked at how mental well-being, harmful drinking, and drinking motivation are represented and interlinked in the UK adult population. The findings reveal a clear association between poor well-being and harmful drinking. Drinking for coping reasons was found to be a significant motivation to drink for many with low mental well-being. Furthermore, it was found that, while mental well-being is directly linked with levels of harmful drinking, the motivation for drinking is a stronger predictor of mental well-being.

Understanding these interrelations between mental well-being, harmful drinking, and drinking motivations may help inform efforts both to identify individuals who could be more effectively supported to reduce their drinking through better mental health and to engage individuals to take steps to reduce their alcohol consumption through the prospect of experiencing higher well-being. In particular, it should be considered whether alternative support and guidance could be provided to reduce the incidence of harmful drinking resulting from people drinking to cope with poor mental well-being. Further research may explore how such interventions could be designed to effectively engage key target audiences and support them to drink in less harmful ways.

## Figures and Tables

**Table 1:** Harmful drinking category (AUDIT) scoring.

Harmful Drinking Categories (AUDIT)	AUDIT Score
Abstainers	0
Low risk	1–7
Hazardous drinking	8–15
Harmful drinking	16–19
Probable dependence	20+

Note: AUDIT: Alcohol Use Disorder Identification Test.

**Table 2:** Well-being category scoring (SWEMWBS).

Well-Being Categories (SWEMWBS)	SWEMWBS SCORE
Low mental well-being	7–19
Medium mental well-being	20–28
High mental well-being	29–35

Note: SWEMWBS: Short Warwick-Edinburgh Mental Wellbeing Scale.

**Table 3:** Proportion of each harmful drinking category that fall into each well-being category.

Well-Being Categories (SWEMWBS)	Harmful Drinking Categories (AUDIT)				
	Abstainers *n* = 747	Low Risk (0–7) *n* = 3424	Hazardous (8–15) *n* = 1515	Harmful (16–19) *n* = 263	Probable Dependence (20+) *n* = 225
Low (7–19)	20.3% _a,b_	14.7% _c_	16.9% _b,c_	22.5% _a,b_	27.3% _a_
Medium (20–28)	68.0% _a_	71.1% _a,b_	73.7% _b_	70.3% _a,b_	63.4% _a_
High (29–35)	11.7% _a,b_	14.1% _b_	9.4% _a_	7.3% _a_	9.3% _a,b_

Note: If a pair of values is significantly different at the 0.05 level, they will have different subscript letters. If a pair of values are not significantly different, the values will have the same subscript letters assigned to them.

**Table 4:** Proportion of drinkers in each well-being category that drink for each drinking motivation.

Well-Being Category (SWEMWBS)	Drinking Motivation (DMQ-R SF)			
	Coping	Conformity	Social	Enhancement
Low (7–19) *n* = 838	72.8%	53.1%	82.7%	82.4%
Medium (20–28) *n* = 3872	59.0%	47.3%	84.9%	83.5%
High (29–35) *n* = 717	35.1%	33.2%	77.0%	76.9%

**Table 5:** Hierarchical multiple regression analysis of predictors of wellbeing.

Predictor Variables	Regression 1	Regression 2	Regression 3
Gender (1, male; 2, female)	0.010	−0.006	0.007
Age (1, 18–24; 5, 55+)	0.184 *	0.172 *	0.146 *
Education level (1, low; 3, high)	0.069 *	0.070 *	0.059 *
Social grade (1, AB; 4, DE)	−0.128 *	−0.133 *	−0.122 *
AUDIT score		−0.078 *	−0.006
Drinking Motivation			
Social reasons (1, yes; 2, no)			−0.048 *
Enhancement reasons (1, yes; 2, no)			−0.056 *
Coping reasons (1, yes; 2, no)			0.228 *
Conformity reasons (1, yes; 2, no)			0.060 *
R^2^	0.054	0.060	0.105
R^2^ Change	0.054	0.006	0.045

Note: Note. Statistical significant: * *p* < 0.001.

**Table 6:** Proportion of drinkers split by drinking motivations and harmful drinking category that fall into each wellbeing category (un-weighted bases reported).

Drinking Motivation (DMQ-R SF)	Well-Being Category (SWEMWBS)	Harmful Drinking Category (AUDIT)			
		Low Risk	Hazardous	Harmful	Probable Dependence
		*n* = 1495	*n* = 1113	*n* = 240	*n* = 200
Coping	Low (7–19)	18.8% _a_	19.9% _a_	24.0% _b_	28.7% _b_
	Medium (20–28)	73.1% _a_	73.6% _a_	69.8% _a,b_	62.4% _b_
	High (29–35)	8.1% _a_	6.5% _a_	6.2% _a_	8.9% _a_
		***n* = 1306**	***n* = 812**	***n* = 172**	***n* = 165**
Conformity	Low (7–19)	17.2% _a_	18.5% _a_	20.3% _a,b_	27.5% _b_
	Medium (20–28)	72.2% _a_	75.9% _a_	72.2% _a,b_	61.4% _b_
	High (29–35)	10.7% _a_	14.1% _b_	7.5% _a,b_	11.1% _a_
		***n* = 2638**	***n* = 1444**	***n* = 248**	***n* = 207**
Social	Low (7–19)	14.8% _a_	16.0% _a, b_	22.3% _b, c_	24.9% _c_
	Medium (20–28)	72.2% _a, b_	74.8% _b_	70.7% _a, b_	65.4% _a_
	High (29–35)	13.0% _a_	9.2% _b_	7.0% _b_	9.8% _a, b_
		***n* = 2533**	***n* = 1464**	***n* = 258**	***n* = 217**
Enhancement	Low (7–19)	14.2% _a_	16.7% _a, b_	22.9% _b, c_	27.2% _c_
	Medium (20–28)	72.4% _a_	74.0% _a_	69.7% _a, b_	63.4% _b_
	High (29–35)	13.3% _a_	9.2% _b_	7.4% _b_	9.4% _a, b_

Note: If a pair of values is significantly different at the .05 level, they will have different subscript letters. If a pair of values are not significantly different, the values will have the same subscript letters assigned to them.
